# Comparison of the Microbiome of Artisanal Homemade and Industrial Feta Cheese through Amplicon Sequencing and Shotgun Metagenomics

**DOI:** 10.3390/microorganisms10051073

**Published:** 2022-05-23

**Authors:** Konstantinos Papadimitriou, Rania Anastasiou, Marina Georgalaki, Rimi Bounenni, Argiro Paximadaki, Christina Charmpi, Voula Alexandraki, Maria Kazou, Effie Tsakalidou

**Affiliations:** 1 Laboratory of Dairy Research, Department of Food Science and Human Nutrition, Agricultural University of Athens, Iera Odos 75, 118 55 Athens, Greece; ranastasiou@aua.gr (R.A.); mgeor@aua.gr (M.G.); rimi.bou@espiv.net (R.B.); paksimadaki@gmail.com (A.P.); christina.charmpi@vub.be (C.C.); valex@aua.gr (V.A.); kmaria@aua.gr (M.K.); et@aua.gr (E.T.); 2 Laboratory of Food Quality Control and Hygiene, Department of Food Science and Human Nutrition, Agricultural University of Athens, Iera Odos 75, 118 55 Athens, Greece

**Keywords:** Feta cheese, 16S rDNA, ITS, shotgun, binning, diversity, ecosystem, lactic acid bacteria, yeasts, microbiota

## Abstract

Feta is the most renowned protected designation of origin (PDO) white brined cheese produced in Greece. The fine organoleptic characteristics and the quality of Feta rely on, among other factors, its overall microbial ecosystem. In this study, we employed 16S rDNA and internal transcribed spacer (ITS) amplicon sequencing, as well as shotgun metagenomics, to investigate the microbiome of artisanal homemade and industrial Feta cheese samples from different regions of Greece, which has very rarely been investigated. 16S rDNA data suggested the prevalence of the *Lactococcus* genus in the homemade samples, while *Streptococcus* and *Lactobacillus* genera prevailed in the industrial control samples. Species identification deriving from shotgun metagenomics corroborated these findings, as *Lactococcus* *lactis* dominated two homemade samples while *Streptococcus thermophilus* and *Lactobacillus delbrueckii* subsp. *bulgaricus* were found to be dominating one industrial sample. ITS data revealed a complex diversity of the yeast population among the samples analyzed. *Debaryomyces*, *Kluyveromyces*, *Cutaneotrichosporon*, *Pichia, Candida,* and *Rhodotorula* were the major genera identified, which were distributed in a rather arbitrary manner among the different samples. Furthermore, a number of potential metagenome-assembled genomes (MAGs) could be detected among assembled shotgun bins. The overall analysis of the shotgun metagenomics supported the presence of different foodborne pathogens in homemade samples (e.g., *Staphylococcus aureus,* *Listeria monocytogenes,* *Enterobacter cloacae,* and *Streptococcus suis*), but with low to very low abundances. Concluding, the combination of both amplicon sequencing and shotgun metagenomics allowed us to obtain an in-depth profile of the artisanal homemade Feta cheese microbiome.

## 1. Introduction

Feta is the most important protected designation of origin (PDO) cheese produced in Greece. It is part of the everyday diet of Greek people, and it is renowned worldwide. Therefore, the production of Feta cheese is an important economic activity for small and medium-sized enterprises (SMEs), as well as large dairy industries aiming to cover the needs of the national market and export demands. 

The production of PDO Feta is regulated by legislation, and it is limited to certain geographic regions within Greece, including Macedonia, Thrace, Epirus, Thessaly, Central Greece, Peloponnese, and the island of Lesvos (Official Government Gazette of the Hellenic Parliament 8/11.01.1994). It should be noted, however, that Feta is produced according to the same rules in the rest of the country, e.g., Crete, albeit not being considered PDO and sold as white brined cheese. Feta is a brined white cheese made from sheep’s milk or a mixture of sheep’s and goat’s milk (30% maximum). The final product must be soft enough so that it can be sliceable (feta means slice in Greek), and it should have a pleasant, slightly acidic taste and rich aroma. Even a superficial examination of the commercially available Feta cheese reveals that, while fitting within the general legislative requirements, it presents a relatively broad range of organoleptic and physicochemical characteristics among the different producers. This is expected, given the differences in milk composition and variations in technology used among the large number of producers (e.g., type of rennet added for milk coagulation, ripening in metallic containers versus wooden barrels, etc.). 

One of the major factors affecting the physicochemical and organoleptic characteristics of Feta cheese is the microbial ecosystem prevailing during production, including the stage of ripening. The microbiology of Feta cheese has been investigated in some instances, and it is well established that the different microorganisms involved undergo a dynamic evolution during its production [1,2]. Similar to many other types of cheese, Feta may contain, among others, starter lactic acid bacteria (LAB), non-starter LAB (NSLAB), and yeasts. Thermophilic LAB, i.e., *Streptococcus thermophilus* and *Lactobacillus delbrueckii* subsp. *bulgaricus,* are often used as starters [3,4,5]. They can be added directly as yogurt, or in other forms (e.g., lyophilized). However, given the importance of rapid acidification for the quality of Feta cheese, mesophilic or mixtures of mesophilic and thermophilic LAB strains are also employed [3,6,7,8]. These would include, among others, strains of *Lactococcus lactis* subsp. *lactis* and subsp. *cremoris*, alone or in combination with *Lb. bulgaricus* [3,4]. It has been suggested that starter populations may decline during ripening due to the low pH (4.4–4.6) and the high salt content of the curd (around 3%) [1,4,9,10,11]. Nevertheless, such conditions seem to favor the growth of lactobacilli, including *Lactiplantibacillus plantarum*, *Lacticaseibacillus paracasei* subsp. *paracasei*, *Levilactobacillus brevis, Lentilactobacillus hilgardii*, *Loigolactobacillus coryniformis* and *Limosilactobacillus fermentum* that are found in the mature product [2,12,13]. These bacteria are practically NSLAB that may exist in raw milk and survive the initial heat treatment, or contaminate the cheese mass and the brine through the environment. Lactobacilli can also be found in the slime generated during the dry salting of Feta. This slime also contains yeasts, and it plays an important role during the late stages of ripening [2]. The main yeast species identified in Feta cheese (including the brine) may include *Candida sake*, *Candida versatilis*, *Candida zeylanoides*, *Candida famata*, *Debaryomyces hansenii*, *Kluyveromyces marxianus*, *Kluyveromyces lactis*, *Pichia farinose*, *Pichia membranifaciens*, *Pichia fermentans*, *Saccharomyces cerevisiae*, *Torulaspora delbrueckii*, *Trichosporon ovoides,* and *Yarrowia lipolytica* [4,12,13,14,15,16,17]. The yeast contamination levels have been suggested to influence the organoleptic characteristics of Feta, which may be positive in general, but yeasts overgrowth may lead to important defects [4,14]. Other microorganisms that may be common in the Feta cheese microbiome are enterococci, pediococci, and leuconostocs that belong to NSLAB [1,2,9]. Additional bacteria, such as coliforms (including *Escherichia coli*), have also been detected in Feta cheese, and their numbers declined during the ripening process [1]. Finally, another aspect that has attracted attention is the use of adjunct cultures (or specific cell extracts) to improve the flavor of Feta cheese [18,19], increase its functional and/or safety properties [20,21,22,23,24], or even accelerate its ripening period [25]. 

Metagenomics is currently the most powerful culture-independent approach for studying food related microbial ecosystems, including the multi-organism environment of traditional cheese [26,27,28,29,30,31]. Amplicon sequencing can indicate which microbial populations occupy a specific niche (mostly at the genus level). Shotgun metagenomics may provide evidence about which microorganisms are present in the niche at a lower taxonomic level (i.e., at the species or subspecies level), but can also reveal their potential functional properties. Amplicon sequencing has been applied in Feta cheese samples before [32,33,34]. However, these studies along with all other studies focusing on the microbiology of Feta cheese relied on commercial, artisanal, or experimental Feta cheese samples produced on an industrial scale or in small-scale cheese making facilities. A search of the relevant literature revealed that the microbial ecosystem in Feta cheese produced in households (i.e., homemade Feta cheese) has rarely been investigated in the past [35,36]. Therefore, we applied for the first time both amplicon-based sequencing and shotgun metagenomics to shed more light on the microbiology of artisanal homemade Feta cheese samples.

## 2. Materials and Methods

### 2.1. Cheese Samples 

Finding artisanal homemade Feta cheese samples proved to be a rather difficult task, since it is not sold commercially, and home producers from different parts of Greece had to be found by word of mouth (viva voce). All samples were transferred to the laboratory at 4 °C, and were stored at the same temperature until analysis. Originally, 11 artisanal homemade Feta cheese samples were selected for analysis. Samples were checked to comply with the typical organoleptic characteristics of Feta cheese by members of the Laboratory of Dairy Research, i.e., samples should have a pleasant taste (acidic and salty) without off-odors or off-flavors while having a soft to semi-hard texture. Several samples presented one or more defects, while preliminary investigation of the extracted DNA from the samples indicated that it was degraded in most of them. Only four homemade samples qualified for subsequent next generation sequencing, exhibiting both correct organoleptic characteristics and intact DNA. Those four samples were obtained from household producers in Agios Stefanos (Attica region, Homemade sample 1), Agrinio (Aetolia-Acarnania region, Homemade sample 2), Aspropyrgos (Attica region, Homemade sample 3), and Heraklion (Crete island, Homemade sample 4). Then, two industrial samples to be used for comparison were purchased from local supermarkets, and were produced by two different established dairy Greek industries located in the Epirus region (Industrial sample 1 and 2). Thus, all samples were produced in locations within the geographic requirements for PDO Feta cheese mentioned above, with the exception of Homemade sample 4, which was included in the analysis as a non-PDO Feta type cheese.

According to the families that produced the homemade Feta cheese samples analyzed, a heating step of milk was applied before the addition of rennet, but the exact parameters of time and temperature could not be specified. In all homemade samples, commercial rennet in liquid form was added except for Homemade sample 2, in which traditional rennet prepared from the stomachs of lambs was used. Furthermore, Homemade samples 1 and 3 were prepared with the use of traditional homemade yogurt made from sheep’s milk, while Homemade samples 2 and 4 were prepared without the addition of any form of starter cultures. Finally, for the two industrial samples, we have to assume that milk was pasteurized, and starters were added as yogurt or in another form (e.g., lyophilized cultures) to initiate the fermentation. All Feta cheese samples analyzed here had been ripened for more than two months, as required by PDO regulations, and were at a stage ready for consumption.

### 2.2. DNA Extraction 

The extraction of DNA from the Feta cheese samples was performed following a modified protocol described previously [37]. Briefly, 0.5 g of Feta cheese were washed with sterile distilled water, and fat was removed after centrifugation. Of note, similar amounts of Feta cheese samples have also been used for DNA extraction by others [32,33]. The pellets were resuspended in phosphate buffered saline (PBS) pH 7.4 and incubated at 65 °C for 10 min to inactivate DNAses that can degrade the extracted DNA. Subsequently, the samples were treated with 600 μL lysozyme (50 mg mL^−1^ in Tris-EDTA buffer pH 8.0), 40 μL mutanolysin (5 U mL^−1^), and 100 μL RNAse A (10 mg mL^−1^) (all purchased from Sigma-Aldrich Chemie Gmbh, Munich, Germany) at 37 °C for 3 h. To further aid cell lysis, 20 μL proteinase K (25 mg mL^−1^) (Sigma-Aldrich) was used, and samples were incubated at 55 °C for 1 h. For the complete denaturation of the samples and the cells’ constituents, 500 μL of the GES reagent, containing guanidium thiocyanate (5 M), EDTA (100 mM), and sarkosyl (0.5%, *v*/*v*) (Sigma-Aldrich), was added. After centrifugation, DNA in the aqueous phase was purified by an ammonium acetate-chloroform extraction, two sequential phenol-chloroform extractions, and a final chloroform extraction. DNA samples were concentrated by 3M sodium acetate (pH 5.2) and cold isopropanol precipitation, followed by washing with 70% *v*/*v* ethanol. The pellet was resuspended in Tris-EDTA buffer pH 8.0. DNA concentration and the 260/280 nm ratio were measured with a Quawell Q5000 Read First spectrophotometer (Quawell Technology Inc., San Jose, CA, USA), while DNA integrity was assessed by agarose electrophoresis. 

### 2.3. Sequencing and Bioinformatics Analysis

For both amplicons (16S rDNA and internal transcribed spacer (ITS)), as well as shotgun metagenomics, library preparation, sequencing and preliminary quality control were performed at Molecular Research DNA (MR DNA, Shallowater, TX, USA) as described previously [38,39]. Data from amplicon sequencing was imported into the CLC genomics workbench 11.0.1 (Qiagen, Hilden, Germany) for chimera removal and operational taxonomic unit (OTU) determination using default parameters. 16S rDNA OTU picking was performed against the Silva database version 138.1 clustered at 99% identity [40]. The Silva database has partially adopted the latest taxonomy for the genus *Lactobacillus* [41] without uniting the *Lactobacillaceae* and *Leuconostocaceae* families. Appropriate comments have been added to indicate this. The relevant analysis of ITS data was performed against the UNITE database version 7.2 at 97% identity [42]. For both 16S rDNA and ITS analysis, we proceeded by selecting settings for a closed OTU picking to decrease the number of unidentified taxa. Rarefying of reads was performed under default settings. Alpha- and beta-diversity of samples were calculated using the total number of OTUs and principal coordinate analysis (PCoA) based on Bray–Curtis distance, respectively. Shotgun reads were initially used for taxonomic profiling of samples, relying on their alignment against a preselected database of bacterial and yeast genomes from GenBank and RefSeq, through the relevant pipeline in the CLC genomics workbench using default parameters. The preselected database of bacterial and yeast genomes is automatically downloaded through CLC genomics workbench, including representative genomes of taxa, and it is available upon request. Rarefying shotgun reads was performed using default parameters and alpha-diversity was calculated using the total number of OTUs. Subsequently, shotgun reads were assembled through the de novo assembly function in the CLC genomics workbench for a minimum length of 1K bp for Industrial sample 1 and Homemade sample 4, while for Homemade sample 2, assembly was performed for a minimum length of 2K bp (please see below). All other parameters were set to default, and the scaffolding option was selected, since it provided better binning results. Assembled scaffolds were further analyzed by BusyBee web server to identify bins and assign taxonomic features to them [43]. Default parameters were employed for the analysis of samples apart from Homemade sample 2, for which all contig length parameters were set to 2K bp and the “min points in neighborhood” was set to 10. Scaffolds from the assembly of shotgun reads were also uploaded to the metagenomics rapid annotation using subsystems technology (MG-RAST) server version 4.0.3 for annotation and functional analysis based on the subsystems source [44]. The heatmap analysis was performed with normalized clustered Euclidean distances of the subsystems categories across the samples using default parameters in MG-RAST. Raw data was deposited to the Sequence Read Archive (SRA) database under BioProject IDs PRJNA774058, PRJNA775995, and PRJNA776012 for samples analyzed by 16S rDNA, ITS, and shotgun metagenomics, respectively. 

## 3. Results and Discussion

### 3.1. 16S rDNA Amplicon Sequencing

The initial analysis of all six Feta cheese samples relied on 16S rDNA amplicon sequencing. The number of sequences and OTUs per sample are presented in Table 1. 

**Figure 1:**
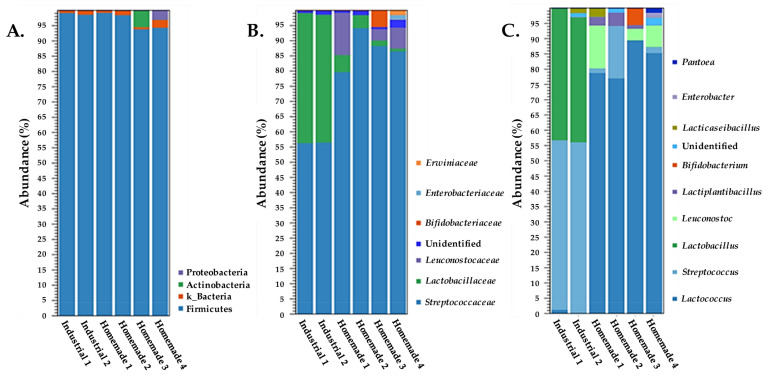
Taxonomic profile of industrial and homemade Feta cheese samples based on 16S rDNA amplicon data. Taxa were clustered at the phylum (**A**), family (**B**), and genus (**C**) levels. Panel (**C**) presents genera with abundance ≥1%.

Figure 1 shows clustering of 16S rDNA reads based on the aggregate features phylum, family, and genus. All samples were dominated at the phylum level by Firmicutes (>94% abundance). Homemade samples 3 and 4 showed as the second larger population Actinobacteria (5% abundance) and Proteobacteria (3% abundance), respectively. An additional population identified at the kingdom level (k_Bacteria) was observed in all samples, demonstrating an abundance of 1%, with the exception of Homemade sample 4, in which it reached 3% abundance. All of these data suggest that the environment of Feta cheese is suitable for the growth and survival of Gram-positive bacteria, mostly Firmicutes and to a lesser extent Actinobacteria. In contrast, Gram-negative Proteobacteria may survive, but do not seem to be able to thrive in this environment, at least not when Feta cheese is prepared correctly.

Industrially produced samples started to clearly differ from homemade ones when compared at the family level (Figure 1B). Industrial samples consisted mainly of *Streptococcaceae* and *Lactobacillaceae,* with similar abundances in both samples (approximately 56% and 43%, respectively). In contrast, the prevalent family for the homemade samples was *Streptococcaceae,* with abundances ranging from 79 to 95%. Members of the *Lactobacillaceae* family were also identified in all homemade samples, but with relatively low abundances (<6%). *Leuconostocaceae* were present mainly in Homemade samples 1, 3, and 4 with 14, 4, and 7% abundance, respectively. Of note, *Leuconostocaceae* has been currently united with *Lactobacillaceae* [41], a change that is not yet reflected in the Silva database. *Bifidobacteriaceae* were evident only in Homemade sample 3, with 5% abundance. Furthermore, two families belonging to Enterobacterales, i.e., *Enterobacteriaceae* and *Erwiniaceae* were detected only in Homemade sample 4, each exhibiting around 1.5% abundance. 

Differences became even more evident when samples were compared for the most prominent genera (≥1% abundance) (Figure 1C). The major member of the *Streptococcaceae* family consisted of the genus *Streptococcus* in the industrial samples, whereas in the homemade ones it comprised the genus *Lactococcus*. Lactococci were also detected in the Industrial sample 1, but with low abundance (1%). Streptococci were also present in Homemade samples 1, 2, and 4, reaching as high as 17% abundance in Homemade sample 2. *Lactobacillus* spp. were restricted in the two industrial samples with 41–43% abundance. In total, two additional genera of the *Lactobacillaceae* family were also detected. *Leuconostoc* spp. were present in Homemade samples 1, 3, and 4 with 14, 4, and 7% abundance, respectively. *Lactiplantibacillus* spp. were detected in Homemade samples 1, 2, and 3 with 1–4% abundance. The genus *Bifidobacterium* exhibited 5% abundance in Homemade sample 3. Of note, while bifidobacteria have been used as probiotic adjunct cultures in cheeses, to the best of our knowledge, the presence of these bacteria in Feta cheese has not been reported before. *Lacticaseibacillus* spp. were identified in Industrial sample 2 and Homemade sample 1 with 2 and 3% abundance, respectively. The Gram-negative genera *Enterobacter* and *Pantoea* were found solely in Homemade sample 4, and they showed around 1.5% abundance each.

Finally, BLASTn analysis of the OTU sequences identified solely as k_Bacteria mentioned above revealed that they corresponded mostly to LAB genera or other Gram-positive bacteria (e.g., *Staphylococcus* spp.). For unknown reasons, these sequences could not be matched to known bacteria.

**Figure 2:**
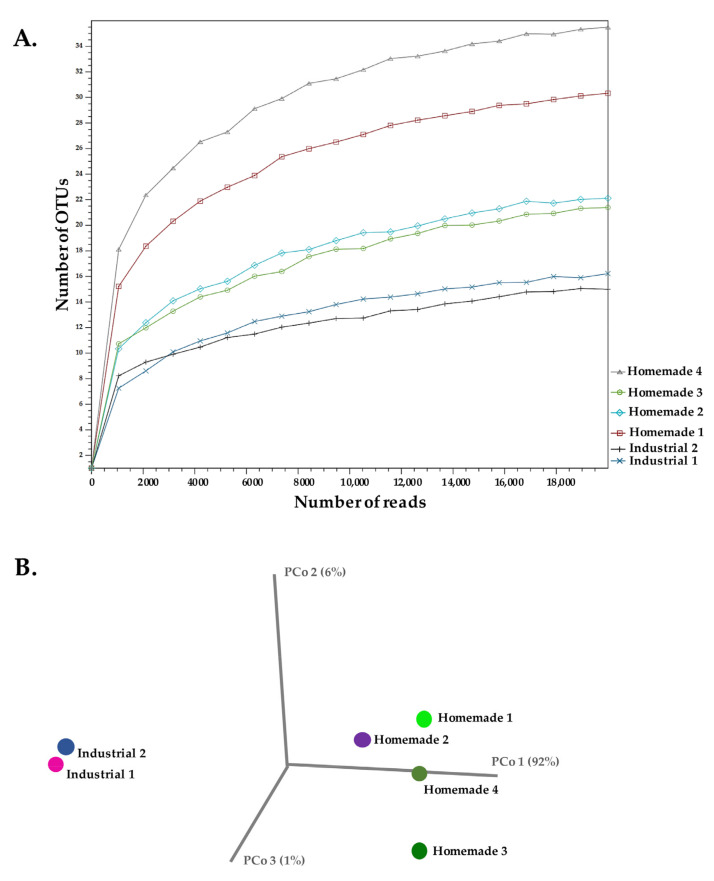
Alpha-diversity analysis of 16S rDNA reads for a maximum depth of 20,000 read counts measured using the total number of OTUs at genus level of industrial and homemade Feta cheese samples (**A**). Beta-diversity presented in a principal coordinate analysis (PCoA) employing the Bray–Curtis distances for the same samples (**B**).

The differences we observed between industrial and homemade Feta cheese samples analyzed up to now are evident. Even though analysis at the phylum level provided a rather uniform picture of all samples, several of the major families or genera deviated in distribution and/or abundance in the two types of samples. Alpha-diversity after rarefying reads and calculating the total number of genus level OTUs detected in each sample suggested that the homemade samples exhibited higher complexity than the industrial ones (Figure 2A). Our findings also indicate that the sequencing depth for the different samples was sufficient in our experiment. In addition, beta-diversity at the genus level demonstrated that the two industrial samples were clustered together and separately from the homemade ones in a principal coordinate analysis (PCoA) using Bray–Curtis distances (Figure 2B). These findings indicate that the two industrial samples had similar microbial diversities as also suggested above (Figure 1). Finally, homemade samples showed some variation in their diversities. They could be separated mainly by the PCo 2 and 3, most probably due to the prevalence of *Lactococcus* spp. in all of them. As mentioned above, one scenario that may be consistent with our results is that the industrial samples were produced with pasteurized milk and starter cultures (yogurt or lyophilized starters). This scenario can be supported given the predominance of the *Streptococcus* and *Lactobacillus* genera, as well as their similar diversities in these samples. In contrast, the prevalence of lactococci in homemade samples indicates that no starters were added or that if yogurt was used for the initiation of the fermentation process, then *Str. thermophilus* and *Lb. bulgaricus* could not dominate the ecosystem of the final product. As mentioned above, Homemade samples 1 and 3 were inoculated with yogurt, but the distribution of *Streptococcus* spp. does not seem to correlate with this fact. Lactococci may have derived from raw milk, especially if the milk was insufficiently heated. Alternatively, they may have contaminated the milk after heat treatment from the environment in which cheese making took place [9]. In addition, it has been previously suggested that ripening conditions could lead to the selection of lactococcal populations [9]. Nevertheless, high abundances of lactococci in industrial Feta cheese samples has also been reported in three recent 16S rDNA metagenomics studies [32,33,34]. The higher richness in identified genera of the homemade samples may indicate lower hygienic conditions prevailing during cheese production when compared to the industrial ones (Table 1 and Figure 2A).

### 3.2. ITS Amplicon Sequencing

**Figure 3:**
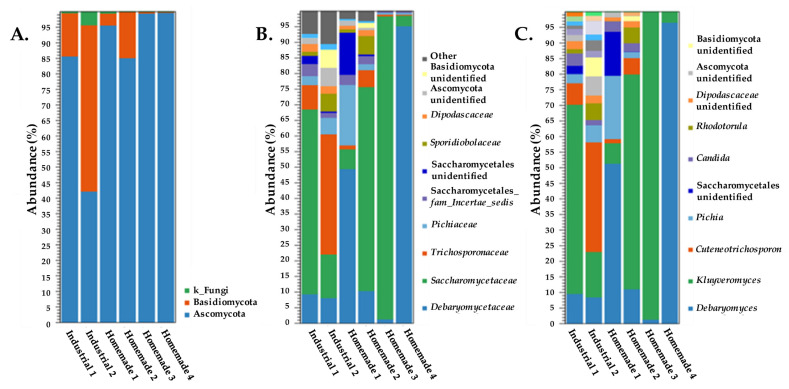
Taxonomic profile of industrial and homemade Feta cheese samples based on ITS amplicon data. Taxa were clustered at the phylum (**A**), family (**B**), and genus (**C**) levels. Panel (**C**) presents genera with abundance ≥1%.

We then analyzed all samples with ITS amplicon sequencing (Figure 3). In most samples, Ascomycota was the predominant phylum with >85% abundance, reaching up to ~99% in both Homemade samples 3 and 4. Industrial sample 2 was the only one exhibiting 42% abundance for Ascomycota, with Basidiomycota presenting the highest abundance (54%). Basidiomycota was the second largest population in all samples. In Industrial sample 1 and Homemade samples 1 and 2, the abundance of Basidiomycota was 14, 4, and 15%, respectively. In contrast, in Homemade samples 3 and 4 Basidiomycota exhibited abundance ≤1%. In the two industrial samples and in Homemade sample 1, an additional population identified at the kingdom level (k_Fungi) was observed, reaching 4% abundance in Industrial sample 2.

Analysis at the family level clearly distinguished Homemade samples 3 and 4 that primarily contained two different Ascomycota families, i.e., *Saccharomycetaceae* (97% abundance) and *Debaryomycetaceae* (96% abundance), respectively. Furthermore, *Saccharomycetaceae* was a major part of the microbiota of Industrial sample 1 (60% abundance) and Homemade sample 2 (65% abundance). The *Debaryomycetaceae* family was also present in all other samples with abundances <11%, with the exception of Homemade sample 1, in which it reached 50% abundance. *Trichosporonaceae* was the most abundant family in Industrial sample 2 (39% abundance), and it was present in reduced populations in all other samples (<8% abundance). Other fungi present in all samples were members of the *Pichiaceae* family and the *Incertae sedis* family of the Saccharomycetales order that reached up to 20% abundance in Homemade sample 1, and 4% abundance in Industrial sample 1, respectively. Additional families distributed in the industrial samples and in the Homemade samples 1 and 2 were detected. The *Sporidiobolaceae* and the *Dipodascaceae* families reached up to 6% abundance in Homemade sample 2, and up to 2.5% abundance in Industrial sample 2, respectively. Additionally, there were three unidentified families either within the Basidiomycota or the Ascomycota phyla. The three unidentified families varied in percentage in the different samples, and only the unidentified family belonging to the Saccharomycetales order exhibited an abundance of 13% in Homemade sample 1. 

**Figure 4:**
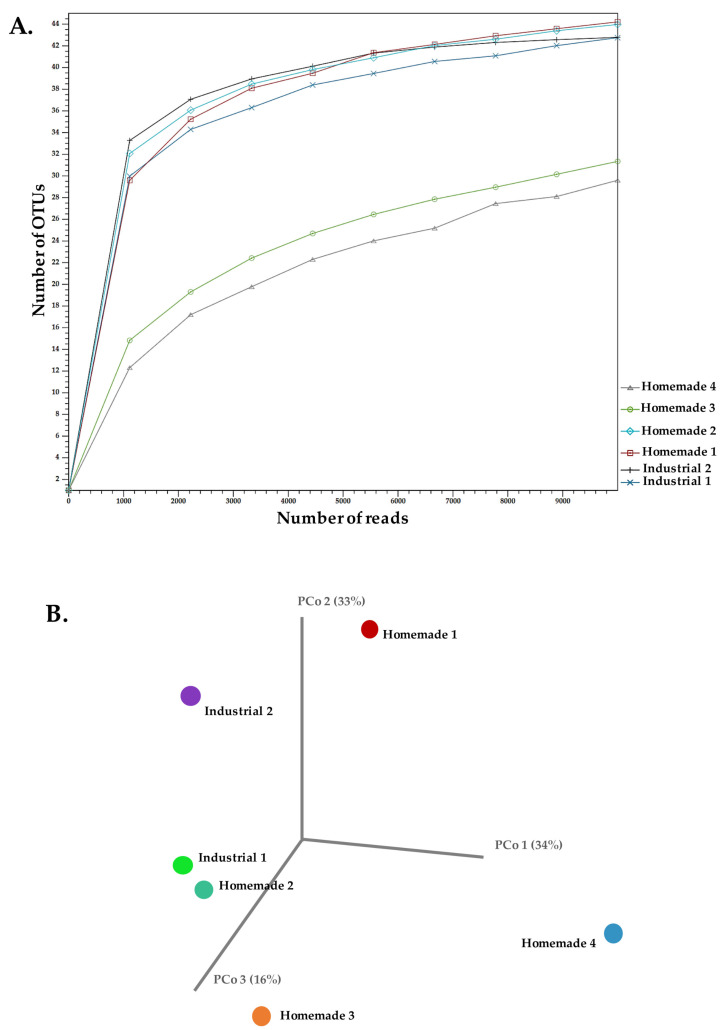
Alpha-diversity analysis of ITS reads for a maximum depth of 10,000 read counts measured using the total number of OTUs at genus level of industrial and homemade Feta cheese samples (**A**). Beta-diversity presented in a principal coordinate analysis (PCoA) employing the Bray–Curtis distances for the same samples (**B**).

When we performed analysis to determine the major genera in the samples (≥1% abundance) (Figure 3C), we found the presence of only one genus for each of the families described above i.e., *Debaryomyces* for *Debaryomycetaceae*, *Kluyveromyces* for *Saccharomycetaceae*, *Cutaneotrichosporon* for *Trichosporonaceae*, *Pichia* for *Pichiaceae,* and *Rhodotorula* for *Sporidiobolaceae*. In addition, the portion of OTUs matched to the family *Incertae sedis* of the Saccharomycetales order was assigned to the *Candida* genus. For the *Dipodascaceae* family, no genus could be identified, and this was the case for the three unidentified families either within the Basidiomycota or the Ascomycota phyla. Thus, most families described in the previous paragraph and their corresponding genera had practically identical abundances. In certain instances, the abundance of genera was below 1%, mostly in Homemade samples 3 and 4, which presented the lowest species richness among all samples (Figure 3C and Figure 4A). The presence of *Debaryomyces*, *Kluyveromyces*, *Pichia* and *Candida* genera in Feta has been reported in a number of studies as mentioned earlier [4,12,13,15,32]. 

Based on the aforementioned results, the yeast diversity in our samples does not exhibit a concise pattern. Alpha-diversity as calculated by the total number of genus level OTUs supported the lower species richness observed for Homemade samples 3 and 4 (Figure 4A). Considering the sequencing depth, all samples clearly reached a plateau after rarefying reads. Beta-diversity based on the genus level revealed a degree of relatedness between Industrial sample 1 and Homemade sample 2 in a PCoA using Bray–Curtis distances (Figure 4B). The rest of the samples were scattered throughout the plot. A similar diversity among yeasts in Feta cheese samples, as judged by ITS amplicon sequencing, was also suggested previously [32]. All of these may indicate that the yeast population in Feta is less controlled by technological steps, suggesting that diversity may arise from raw material, the brine and/or the environment in which the production takes place. These routes for yeast inoculation/contamination of the final product have been suggested before [45].

### 3.3. Species Identification Using Shotgun Metagenomic Sequence Reads of Feta Cheese Samples

**Figure 5:**
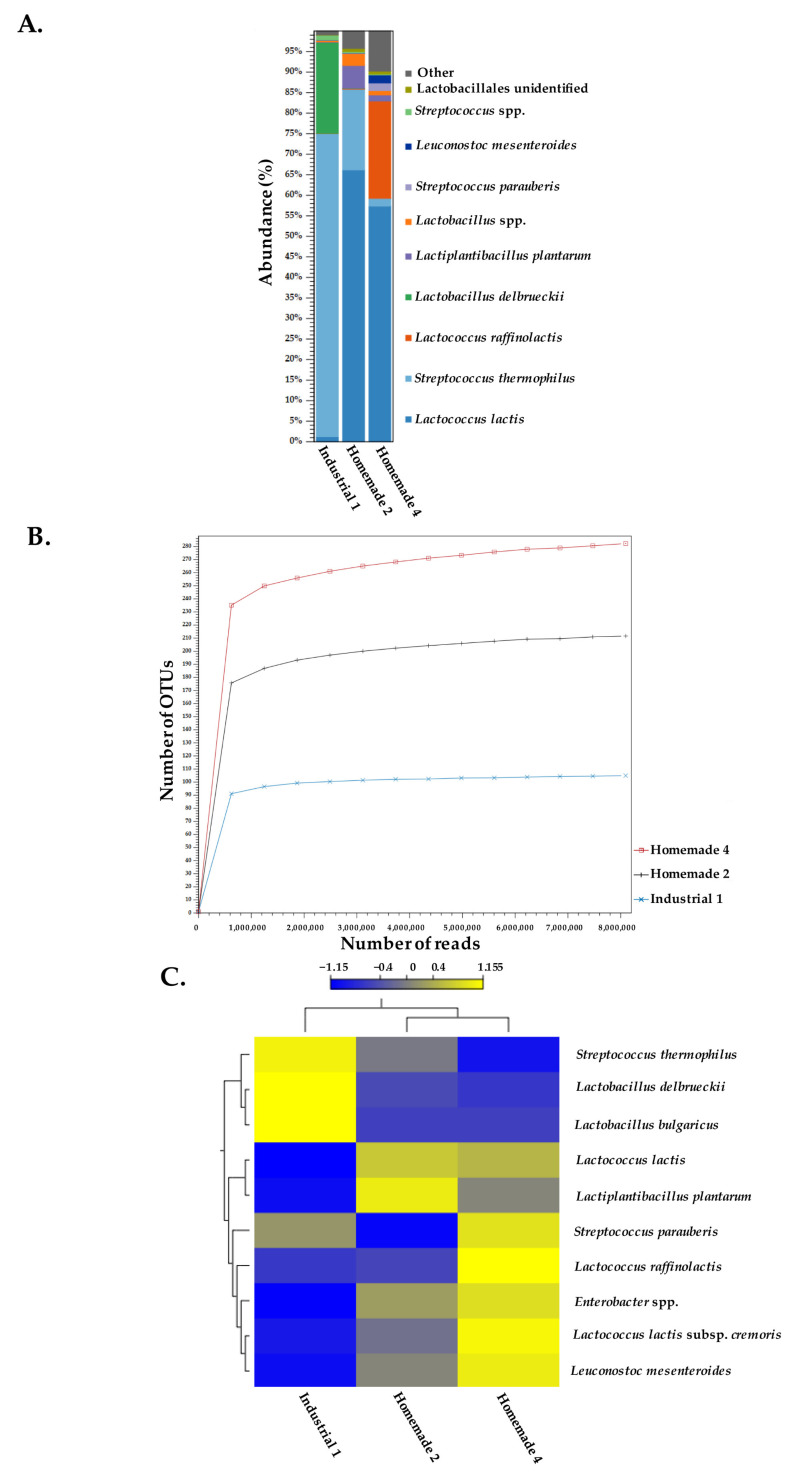
Taxonomic profile of Industrial sample 1 and Homemade samples 2 and 4 of Feta cheese, based on the mapping of shotgun metagenomics reads against a representative genome dataset deriving from GenBank and RefSeq. Taxa were clustered at the species level (**A**). Alpha-diversity of shotgun reads measured using the total number of OTUs of industrial and homemade Feta cheese samples. In the graph, 8M reads are shown (**B**). Heat map analysis of the 10 most abundant species/subspecies present in at least two samples (**C**).

We proceeded with species level identification within the Feta cheese microbiome using shotgun metagenomics. Among the six samples analyzed above, one industrial and two homemade samples were selected for the shotgun metagenomics analysis. Only one industrial sample was selected, given that the two industrial samples had very similar microbiomes at least for the bacterial populations (Figure 1C). Homemade samples 2 and 4 were selected, since they were produced by spontaneous fermentation and not by the traditional practice of adding yogurt, allowing for the possibility of discovering more diverse microorganisms in Feta cheese. Reads from these samples were searched against a representative database of bacterial and fungi genomes (Figure 5A). Species level analysis demonstrated that Industrial sample 1 was characterized by the presence of *Str. thermophilus* (74% abundance) and *Lb. delbrueckii* (22% abundance). This *Lb. delbrueckii* reference sequence derived most probably from an uncharacterized *bulgaricus* subspecies (please see below). In contrast, in Homemade samples 2 and 4, *Lc. lactis* was the most prevalent species, with abundances of 67 and 58%, respectively. The second largest population in Homemade sample 2 was *Str. thermophilus* with 20% abundance, while in Homemade sample 4 it was *Lactococcus raffinolactis,* with 24% abundance.

These findings are in accordance with the aforementioned results of the 16S rDNA amplicon sequencing (Figure 1C), and shed more light into the biodiversity of the Feta cheese microbial ecosystem. In addition, alpha-diversity of rarefied shotgun reads suggested that bacterial diversity was higher in the homemade samples compared to that of the industrial sample (Figure 5B). This is also in accordance with the 16S amplicon data (Figure 2A). Heat map analysis using the 10 most prominent species/subspecies within the three samples established that the bacterial diversities in Homemade samples 2 and 4 were closer than that of Industrial sample 1 (Figure 5C). Among these bacterial species, it is worth mentioning the alignment of sequencing reads to two different *Lb. delbrueckii* genomes (one subsp. *bulgaricus*) in Industrial sample 1 and the alignment of sequencing reads, especially of Homemade sample 4, to a *Lc. lactis* subsp. *cremoris* genome. In addition, the presence of *Lpb. plantarum* and *Leuconostoc mesenteroides* was also evident; the first found mostly in Homemade sample 2 (5% abundance), and the second in Homemade sample 4 (2% abundance). Furthermore, *Enterobacter* sp. was also detected primarily in Homemade sample 4, showing 1% abundance. 

Additional LAB species (e.g., different lactobacilli, *Enterococcus* spp., *Pediococcus* spp., *Weissella* spp., etc.), as well as non-LAB species (*Staphylococcus* spp., *Macrococcus* spp., *Listeria* spp., *Escherichia* spp., *Pseudomonas* spp., *Moraxella* spp., *Enterobacter* spp., etc.), were also identified, and were distributed differently among the samples analyzed. In all cases, the abundance of these taxa was low to very low (well below 0.3%). Within these genera, some foodborne pathogens like *Staphylococcus aureus* (0.3% abundance) and *Listeria monocytogenes* (up to 0.04% abundance) were identified only in the two homemade samples. Both foodborne pathogens and spoilage bacteria can be found in raw milk or the cheese processing environment, and their low to very low abundances in the cheese samples analyzed show that they could not grow under the conditions prevailing in Feta cheese. This is also supported by previous studies testing the growth and survival of foodborne pathogens and/or spoilage bacteria in Feta cheese, such as *L. monocytogenes* [46,47], *E. coli* [1,48], *St. aureus* [49], *Yersinia enterocolitica* [50], and *Salmonella* Enteritidis [51]. It should be mentioned that the low abundance of many of these bacteria may also indicate that they were non-viable in the samples. The fact that these bacteria were identified solely in homemade samples could be attributed to the hygienic conditions during milk and cheese production. We should highlight the identification of *Streptococcus parauberis* in all samples analyzed, a bacterium mostly associated with dairy cattle mastitis [52], which was also found previously in a sample of industrial Feta cheese [33]. 

Finally, it is important to state that none of the reads in our dataset had hits against yeast genomes. This could be explained either by a very low yeast population in our samples that were not represented in the shotgun metagenomics reads or by the inclusion of inadequate yeast genomes in the dataset that failed to match the diversity of our samples. The latter is not the case, since several genomes of the genera *Debaryomyces* (e.g., *D. hansenii*)*, Kluyveromyces* (e.g., *K. lactis* and *K. marxianus*), *Pichia,* and *Candida* were present in the reference genome dataset, but received no hits. 

### 3.4. Binning Metagenomic Scaffolds of Feta Cheese Samples

**Figure 6:**
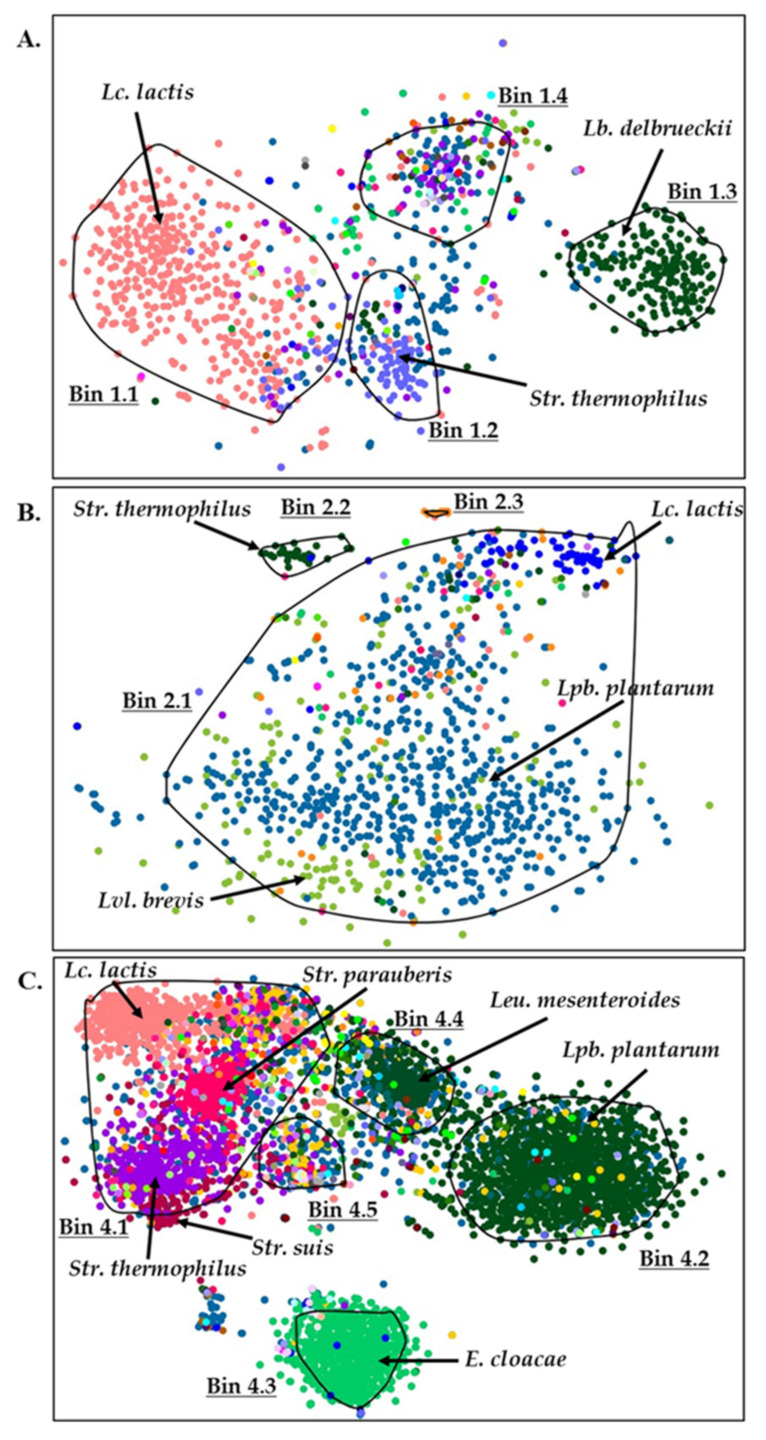
Bins of metagenomics scaffolds of Industrial sample 1 (**A**) and Homemade samples 2 (**B**) and 4 (**C**) of Feta cheese. Dots of same colors represent scaffolds that derived from the same species which are presented within the figure.

We then proceeded with the binning of scaffolds produced from the assembled reads to see if we would be able to identify potential metagenome-assembled genomes (MAGs) (Figure 6). In Industrial sample 1, three bins were almost complete (completeness > 90.0%), and one was partially complete (82.0% completeness) (Table 2). Bins 1.2 and 1.3 exhibited low contamination (6.3% and 1.8%, respectively). Bins 1.1 and 1.4 exhibited higher contamination (19.8% and 18.0%, respectively) containing parts of diverse microorganisms (43.5% and 20.0% strain heterogeneity, respectively). Taxonomic analysis of the bins suggested that bin 1.1 corresponds to a *Lc. lactis* genome, bin 1.2 to a *Str. thermophilus* genome, bin 1.3 to a *Lb. delbrueckii* genome, while no clear identification could be achieved for bin 1.4 (Figure 6A). 

BLASTn analysis of random scaffolds from bin 1.3 suggested that they derived from a *Lb. delbrueckii* subsp. *bulgaricus* (identity > 98%). In Homemade sample 2, assembly and binning parameters had to be adjusted so that bins could be formed. Assembly was performed for minimum 2000 bp fragments, and binning parameters were adjusted as discussed above. In Homemade sample 2, bins 2.1 and 2.2 were almost complete (completeness > 95.0%). Bin 2.1 exhibited high contamination levels (103.6%) containing parts of diverse microorganisms (strain heterogeneity 4.6%). Taxonomic analysis of bin 2.1 corresponded to genomes of *Lpb. plantarum*, *Lvl. brevis* and *Lc. lactis* (Figure 6B). In contrast to bin 2.1, bin 2.2 corresponded to a single genome of *Str. thermophilus* (0.9% contamination). Bin 2.3 could not be assigned to a meaningful group of sequences (0% completeness). In Homemade sample 4, bins 4.1, 4.4 and 4.5 were almost complete (completeness > 92.0%). Bin 4.1 exhibited high contamination levels (232.4%), containing parts of diverse microorganisms (strain heterogeneity 21.0%). Taxonomic analysis of this bin corresponded to genomes of *Lc. lactis, Str. thermophilus, Str. parauberis* and *Streptococcus suis* (Figure 6C). Bins 4.4 and 4.5 showed 9.0% and 4.5% contamination, respectively.

The main genome sequences within bin 4.4 could be identified as *Leu. mesenteroides,* while for bin 4.5, no major assignment could be made. Bin 4.2 was found partially complete (75.7% completeness) with important levels of contamination (35.1%), nevertheless the majority of scaffolds corresponded to a *Lpb. plantarum* genome. Similarly, most sequences in bin 4.3 could be related to the genome of *Enterobacter cloacae,* despite the fact that the bin was found to be incomplete (completeness < 50.0%). In all samples analyzed, genomic parts of different bacterial species (mostly LAB) were identified, dispersed across the 2-D scatter plot. Findings of the binning process suggested the presence of MAGs in certain instances, but this was not always the case. Taxonomic analysis of bins was in overall agreement with the 16S rDNA metagenomics analysis, but perhaps most importantly with the species identification through the alignment of shotgun reads to reference genomes described in the previous paragraph. For example, in Industrial sample 1, bin 1.1 consists mostly of *Lc. lactis* scaffolds, even though this species appears with low abundance in the sample (1.2% abundance). Similarly, bin 2.1 shows several scaffolds of *Lvl. Brevis,* which exhibited 0.7% abundance in Homemade sample 2. Scaffolds of *Str. suis* are also evident in bin 4.1, even though this species shows 0.1% abundance in Homemade sample 4. Of note, *E. cloacae* in Homemade sample 4 is an opportunistic pathogen, exhibiting sometimes multidrug resistance [53], which has been identified in different cheeses [54,55,56,57]. Furthermore, *Str. suis* also present in Homemade sample 4 has been identified in some instances in different types of cheese [58,59], and it has been characterized as an emerging zoonotic pathogen [60]. In addition, *Str. parauberis* has been identified in raw bovine and ovine milk [61,62], artisanal sheep’s and goat’s milk cheese made in Italy [63], as well as a sample of industrial Feta cheese [33]. The current version of Busy-bee uses Kraken (v0.10.5-beta) for taxonomic annotation in combination with the Minikraken database constructed from complete bacterial, archaeal, and viral genomes in RefSeq [43], and thus it cannot predict the presence of yeasts in the microbiomes described above.

**Figure 7:**
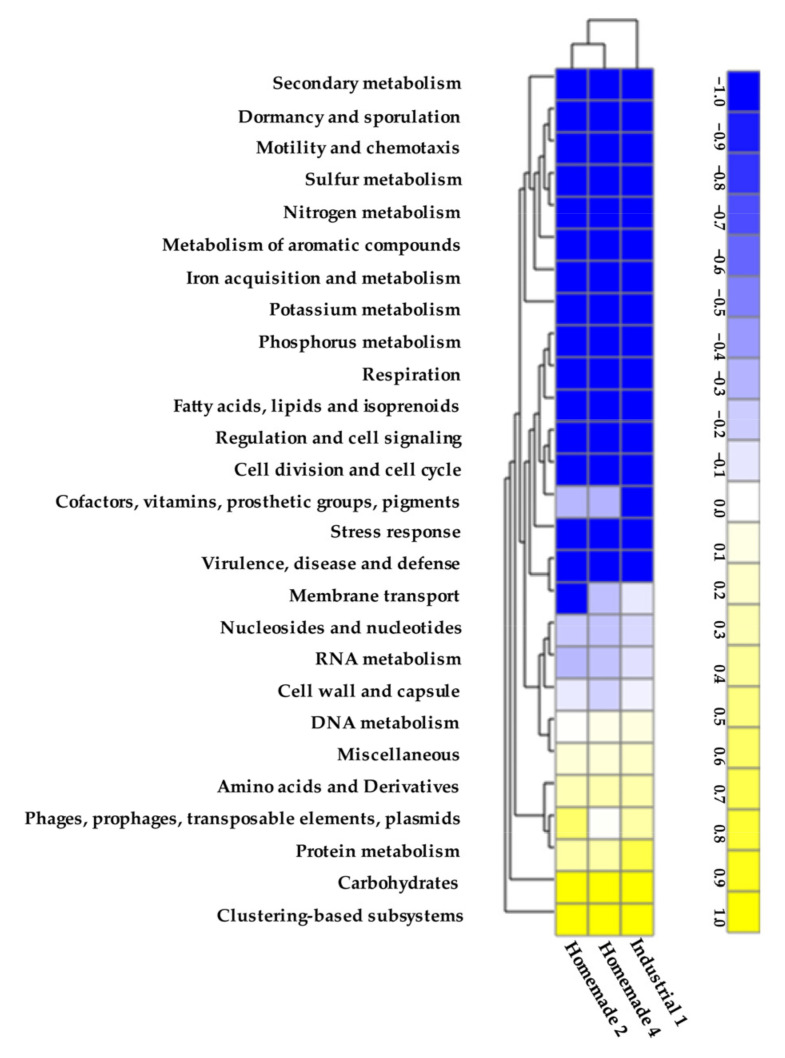
Functional analysis of annotated scaffolds of Industrial sample 1 and Homemade samples 2 and 4 of Feta cheese. Heat map analysis of subsystems categories as calculated by MG-RAST.

We finally performed functional analysis of the assembled shotgun metagenomes with MG-RAST (Figure 7). This is an important step in the analysis of shotgun metagenomics data since the pathways identified within the microbiomes may underly desirable or undesirable properties of cheese [31]. Scaffolds were annotated and assigned to subsystems categories. Heat map analysis supported again the fact that Homemade samples 2 and 4 were more similar, while Industrial sample 1 was distinct. This may reflect the fact that the prevailing species in Homemade samples 2 and 4 were common to a degree, especially *Lc. lactis*, influencing the gene content of the relevant metagenomes. It should be noted that representative genes were identified practically in all most functional categories, as also reported previously in similar cases [64,65,66,67]. This is more or less expected, given that shotgun metagenomics provide an overview of the metabolic and no-metabolic functions encoded in the genomes of the microorganisms of a given eco-system. Some noticeable differences among the three samples could be related to the cofactors, vitamins, prosthetic groups, pigments subsystem, the membrane transport subsystem, and the phages, prophages, transposable elements, and plasmids subsystem. Even though these functions may be important from a technological perspective, e.g., the ability to synthesize co-factors or vitamins or to resist phages, more investigation is required to assess their influence at the species or microbiome level. An additional explanation about these differences would be that these subsystems do not refer to housekeeping processes and thus they may be relatively variable in the three microbiomes. However, such an explanation may not be definitive, and further research is required.

## 4. Conclusions

In this study, we employed 16S rDNA and ITS amplicon sequencing followed by shotgun metagenomics to investigate the microbial ecosystem of artisanal homemade Feta cheese. As expected, amplicon sequencing provided us with a detailed picture of bacteria and yeasts present in the samples, reaching up to the genus level. Our data clearly supports important differences between industrial and homemade samples. Based on shotgun metagenomics, industrial Feta cheese was dominated by *Str. thermophilus* and *Lb. delbrueckii* subsp. *bulgaricus,* while homemade samples by *Lc. lactis*. This is a major difference that may influence both physicochemical properties and organoleptic characteristics of the final product. It has been previously suggested that starter bacteria survive only for a few days after the completion of fermentation, and that NSLAB (mostly lactobacilli) prevail until the end of the ripening due to low pH and increased salt content [1,4,9]. This hypothesis is not entirely verified by our findings. Starter *Str. thermophilus* and *Lb. bulgaricus* reached at least 96% abundance in Industrial sample 1. This fact may indicate that, under good hygienic conditions, competing microflora is extinguished, allowing the prevalence of the two yogurt bacteria during ripening. The high lactococcal populations in the homemade samples may be the result of the production hygiene, but they may also be favored by the conditions during ripening. For example, yogurt was used to start the fermentation in Homemade samples 3 and 4, but lactococci were still dominant in the final product. However, we also hypothesize that temperature at which ripening takes place may also affect the population that will finally dominate. Ripening in households may take place at higher temperatures than in the dairy plant. Given the mesophilic nature of *Lc. lactis*, these conditions may allow residual growth in homemade samples. In contrast, in industrial samples, lower temperatures may protect the thermophilic yogurt bacteria under the harsh ripening conditions, preventing, at the same time, the growth of *Lc. lactis*. It should be mentioned that NSLAB (mostly lactobacilli) were detected in the homemade samples, but their abundance was restricted in all cases. Overall, the systematic dominance of *L. lactis* in all homemade samples besides all possible NSLAB could not have been anticipated, and it constitutes an important difference between industrial and homemade samples. Our findings are also supported by the finding reported about household Feta cheese demonstrating a frequent abundance of lactococci [35,36]. Alpha-diversity of the microbiomes was higher in the homemade samples, reinforcing the hypothesis that hygienic conditions during homemade cheese production were poorer than those of the industrial ones. Among the different LAB and non-LAB genera/species identified in the homemade samples (especially Homemade sample 4), there were some foodborne pathogens. In all cases, the abundance of these genera/species was very low, raising questions about their viability.

In contrast to the bacterial composition of the Feta cheese samples, yeasts were clearly distributed in a rather arbitrary manner. As mentioned above, this finding shows that conditions during production of Feta cheese may not be sufficient to exert selective pressures for the growth of specific yeast species. This is in accordance with previous studies suggesting that yeasts found in Feta cheese are characteristic for the cheese making plant [13]. Unfortunately, an analysis of shotgun metagenomics data did not allow identification of yeasts, presumably due to their low abundance in comparison to the bacterial population.

Binning of scaffolds from assembled shotgun reads resolved a number of sequence bins some of which could be assigned to potential MAGs of variable completeness and quality. Species identification of bacteria was more or less in agreement with the results obtained from the analysis of the unassembled shotgun reads. To the best of our knowledge, metagenomics analysis allowed us to identify *Bifidobacterium* spp., *Cutaneotrichosporon* spp., *E. cloacea* and *Str. suis* in Feta cheese for the first time. The presence of some foodborne pathogens indicates that production of cheese in households should be more cautious. As mentioned above, the information obtained from homemade producers derived during our communication with the producers. Details about heat processing and ripening temperature may be relative; nevertheless, information about the addition of yogurt or not as a starter culture may be more trustworthy. In all such cases, the only thing that can be carried out is to hypothesize the critical points in the production procedure which may explain the results obtained. This retrospective evaluation of the putative factors which may support our findings is not uncommon, especially when it is not practically possible to properly monitor the exact details of the production procedure. Unfortunately, such uncertainty may be inherent in the analysis of homemade samples. It should be emphasized though that the characteristics of the microbiome of the homemade samples seem to be systematic, indicating that our observations are not random. Our findings may also have implications for the starter cultures employed to produce industrial Feta cheese. Given that the homemade samples analyzed had typical organoleptic characteristics of Feta cheese, they may offer the possibility to isolate alternative *L. lactis* starter strains. Such strains may be tested as the sole starters for Feta cheese production, beyond the typical *S. thermophilus*/*Lb. bulgaricus* combinations, to aid the diversity of the product. Homemade Feta may constitute an alternative reservoir for the discovery of novel starter strains. Isolates from the homemade samples are under investigation for their technological and probiotic potential. Lastly, our study shows that the combination of amplicon and shotgun metagenomics is a powerful tool for the analysis of the microbiomes of fermented foods such as Feta cheese, and can lead to the identification of microorganisms that would be missed by other culture-dependent or culture-independent methods.

## Figures and Tables

**Table 1:** Number of reads and OTUs for 16S rDNA and ITS amplicons of the Feta cheese samples.

	16S rDNA Amplicon		ITS Amplicon	
Samples	Number of Reads *	Number of OTUs	Number of Reads *	Number of OTUs
Industrial 1	34,683	19	31,811	49
Industrial 2	32,556	19	12,026	44
Homemade 1	22,334	33	25,127	48
Homemade 2	24,466	26	50,218	50
Homemade 3	37,619	26	134,137	45
Homemade 4	25,693	41	280,150	50

* Numbers refer to reads assigned to OTUs at the genus level after quality control, removal of chimeric sequences, and taxonomic assignment.

**Table 2:** Quality of bins obtained from shotgun metagenomics of the selected Feta cheese samples.

Sample	Bin	Completeness *	Contamination *	Strain Heterogeneity *
Industrial 1	1.1	82.0	19.8	43.5
	1.2	90.1	6.3	14.3
	1.3	95.5	1.8	50.0
	1.4	93.7	18.0	20.0
Homemade 2	2.1	95.5	103.6	4.6
	2.2	95.5	0.9	0.0
	2.3	0.0	0.0	0.0
Homemade 4	4.1	95.5	232.4	21.0
	4.2	75.7	35.1	65.2
	4.3	47.8	3.6	25.0
	4.4	92.8	9.0	0.0
	4.5	93.7	4.5	40.0

* as calculated by the BusyBee web server.

## Data Availability

The data presented in this study are openly available in SRA under BioProject IDs PRJNA774058, PRJNA775995 and PRJNA776012.
